# A Retrospective Analysis of Familicide in Latium (Italy): A Criminological Profile of the Victims and Offenders Involved in 29 Cases and a Comparison with the Literature

**DOI:** 10.3390/healthcare11172448

**Published:** 2023-09-01

**Authors:** Alessandro Mauro Tavone, Giulia Ceccobelli, Giorgia Piizzi, Maria Chiara Clericò, Raimondo Vella, Naomi Romaniello, Gabriele Giuga, Saverio Potenza, Gian Luca Marella

**Affiliations:** 1 Department of Biomedicine and Prevention, University of Rome Tor Vergata, 00133 Rome, Italy; alessandromauro.tavone@students.uniroma2.eu (A.M.T.); raimondo.vella@students.uniroma2.eu (R.V.);; 2 Department of Surgical Sciences, University of Rome Tor Vergata, 00133 Rome, Italy; gian.luca.marella@uniroma2.it

**Keywords:** forensic pathology, familicide, victim/offender relationship, domestic violence, domestic homicide

## Abstract

Familicide, an extreme form of domestic violence where one family member kills another, is a complex criminological issue. We analyzed autopsy files from the Institute of Legal Medicine, University of Rome Tor Vergata (1995–2022), to understand familicide better. The study focused on victim profiles, offender characteristics, and case dynamics. From 29 analyzed cases, 31 victims emerged, with 2 instances of double homicide. The perpetrators were mostly male (79.31%) and the victims were primarily female (54.84%). The familial ties ranged from parent–child to siblings and spouses. A significant number of crimes happened at private residences (70.97%) using bladed weapons (48.39%), with the injuries being concentrated on the head and chest. Half of the cases showed struggle signs, and 24.14% of the perpetrators had identifiable psychiatric disorders, which often served as the motive. Post-crime actions included self-reporting, suicide attempts, and successful suicides. A comparison with literature confirmed the typical familicide offender as a middle-aged male with potential social stressors and a history of domestic violence, with the victims often being female family members. Mental health conditions including depression, bipolar disorder, and schizophrenia significantly impacted these events. These findings underline the need for customized approaches to comprehending and preventing familicide.

## 1. Introduction

From a broad perspective, the family is considered as a human community, which is characterized differently in various historical and geographical contexts, but generally consists of individuals linked by cohabitation, kinship, and affinity. This constitutes a fundamental element of every society, as its processes and relations aim at the perpetuation of the species through reproduction [1].

From a legal perspective, in Italy, with the enactment of the Cirinnà Law, the family unit has extended its definition to include “people connected by cohabitation, kinship, affinity” [2].

The family represents the primary environment in which an individual is placed, an environment that tends to remain throughout the individual’s life journey.

The relationship established with family members provides most of the tools and fundamental principles for inclusion within the social community [3]. The family’s educational project requires harmonious relationships among all its members and a balanced growth of their personalities.

Given that the family should ideally represent the place of utmost safety, when it becomes the scene of heinous violence, it arouses particular interest from criminological, psychiatric, legal, and forensic medical standpoints.

Homicide within the family accounts for a significant percentage of homicides. The EU.R.E.S. [4] (Economic and Social Research), in its report on homicides in Italy, identified the family as the main setting where homicidal crimes occur.

It is notable that 51.5% of homicides take place within the family, which, with 35% of total victims, is confirmed as the primary setting for homicides. For instance, in 2002, out of 223 intra-family homicides, female victims predominated, accounting for 63.2% of cases compared to 36.8% of males—with higher numbers in the north and center, while in the south, the divergence index was significantly reduced.

Family victims predominantly fall within the age group from 25 to 34 years (22.4%); the number of victims up to 18 years old (including infanticides) is significant, standing at 13.5%.

Female victims are higher in number, predominantly due to crimes of passion, which constitute the primary motivation for domestic homicides. The main perpetrators of passion-driven homicides are men, while female killers often suffer from mental disorders.

Furthermore, cases where parents kill their children are significant, with the prevalent motivation being linked to adolescent maladjustment, or reasons related to family disputes arising from the redefinition of relationships and roles between spouses and children.

In cases where the child kills the parent, in addition to economic reasons, situations of high conflict characterized by children’s claims towards the family or the external environment also assume significance.

Intrafamily communication problems also play their part in this phenomenon: parents ignore their children’s difficulties, becoming a less and less stable reference point in managing the difficulties encountered with the external world, work, and affections [5].

This study examines the phenomenon based on the case history from the Institute of Forensic Medicine at the University of Rome “Tor Vergata”, aiming to provide a criminological profile of the actors involved in family homicide.

## 2. Materials and Methods

For our study, we conducted a review of all the autopsy files carried out at the Institute of Legal Medicine at the University of Rome Tor Vergata during the period from 1995 to 2022. By consulting the medico-legal technical consultancy reports and information present in the investigation files, all the files unrelated to homicide cases were excluded in a first screening. For the purpose of this investigation, the definition of family was assumed as “a human community, differently characterized in various historical and geographical situations, but generally formed by people linked to each other by a cohabitation, kinship, affinity” [1], thus also including the category of so-called “de facto couples”, recognized as a family unit in relatively recent times (Law “Cirinnà” n. 76/2016) and made up of people who, despite living together stably and being linked by a long-lasting emotional relationship, have decided not to formalize their union in the municipality, which instead is characterized as “de facto cohabitants”.

Therefore, for the selection of eligible cases, the inclusion criterion was: “all cases of homicide in which there was certainty of the family link, as above understood, between the subjects involved, including de facto unions or stable cohabiting partners, even when the love relationship had recently ended.” Cases were excluded when a culprit had not been identified with certainty, autopsy reports were incomplete, and circumstantial information was absent. Therefore, 29 cases were included.

A data extraction sheet was created in a Microsoft Excel spreadsheet for the construction of two databases, one for each of the two protagonists involved in the murder (victim/offender). The following information regarding the victim was collected:

Age; sex; employment; victim/offender relationship; location of victim discovery; type of injury mechanism; total number of injuries; presence/absence of signs of struggle; and location of injuries.

Regarding the perpetrators of the murders, information was collected relating to:

Age; sex; employment; offender/victim relationship; presence/absence of psychiatric pathology; and motivation for the crime.

Descriptive statistics used frequencies and percentages to describe the demographic, circumstantial, and injury characteristics of the subjects. For the description of the quantitative variables (age and number of injuries), mean values and standard deviations were used.

## 3. Results

The results of the analysis of our case history are summarized in Table 1, Table 2 and Table 3.

In the case series examined, consisting of 29 homicide cases, the total number of victims was 31, considering the presence of 2 double homicide cases. Almost all the victims were adults: in one case, the victim was a male newborn, killed immediately after birth, in one case, the male victim was a few months old, and in one case, the female victim had just turned one year old. Considering the role of the victim within the family, it was observed that: the total number of offenders present in the case series we considered was 29, all having been identified. For the 29 cases analyzed, in only 2 of these were two victims present at a single crime scene. In all 29 cases, the perpetrator acted alone, committing in two cases, a double homicide.

From the anamnestic information concerning the personal history of the offenders, it emerged that seven of them (24.14%) had been diagnosed with a psychiatric disorder. Of these seven cases, in six, the psychiatric disorder was found to be the motivation behind the homicide (in one specific case, the offender had auditory hallucinations at the time of the commission of the fact). Of the seven perpetrators affected by psychiatric pathology, six were male, while in a single case, the offender was female. The psychiatric pathologies under consideration related to affective psychosis, paranoid schizophrenia, hyperactivity, learning disorder, delusions with themes of unworthiness and guilt, depression, anxious-depressive syndrome, and paranoid psychosis.

A previous criminal history was found in two cases, one involving brawl and abuse, and a second case with the offender having been previously sentenced to 3 months imprisonment for the attempted murder of a cohabitant.

Regarding alcohol or drug abuse, out of the 29 cases, 3 were found to have alcohol abuse, while in a single case, both alcohol abuse and drug abuse were present; for five offenders, the abuse of one or other substance was present, but not expressly specified.

From the anamnestic and testimonial information, it was observed that, following the commission of the crime, in six cases, the offender decided spontaneously to self-report. In three cases, the offender was found about to commit suicide just after the murder, and in two cases, they committed suicide in the subsequent moment to the commission of the act.

## 4. Discussion

Homicide within the family, or familicide, represents a harrowing and profound manifestation of domestic violence that poses complex questions for criminologists.

In the current study on familicide, we opted for a family definition that encompasses a broader sample than what the traditional definitions in the literature typically allow. While the widely accepted definition by Wilson, Daly, and Daniele [6] from 1995 describes familicide as “the killing of a spouse and one or more children,” it is evident that this definition may now be outdated. It potentially excludes cases that, despite not strictly adhering to legal, religious, or “traditional family” constructs, exhibit pathological, forensic, and psychiatric features highly relevant to the context of familicide. The definition we adopted, derived from the sources mentioned in the materials and methods [1,2], ensures that discussions on familicide include cases where the bonds, emotions, and interpersonal relationships characteristic of a family align with the earlier definition. However, it acknowledges that these relationships might not be limited to just spouses and children, making this approach more reflective of today’s societal dynamics.

According to the literature, familicide victims span across all age groups, although children [7] and women [8] appear most frequently. Male victims are not uncommon, but are less represented compared to females. We also observed this trend across the victims’ sexes in our cases, but without great differences (54.84% F vs. 45.16% M) Child victims range from very young (infants and toddlers) to adolescents, often depending on the children’s ages within the offending family. In our groups, children represented 12.90% of the victims, with three of them being under 1 year of age.

The victims in familicide cases often share close relationships with the offenders—they are spouses, offspring, or other close family members [9], as it favors the victims being perceived as sources of the offender’s distress [10]. In our cases, the most common relationship between the victims and perpetrators was that of spouses, accounting for 22.58% of the cases. Parents featured prominently in both victim and perpetrator roles (16.13% and 19.35%, respectively), as did children (19.35% victims, 16.13% perpetrators). In certain cases, revenge against an estranged spouse may be a motivation for the killing of children [11].

Siblings, cohabitants, and other relationships such as uncles, nephews, grandparents, son-in-law, father-in-law, and brother-in-law also appear, but are less common. Familial dynamics, dependency relationships, and potential histories of domestic abuse significantly influence the victim profile in these cases [12].

In our cases, the home of the victim, shared with the offender, was the predominant scene of the crime. Different authors have suggested that shared living spaces between victims and offenders represent a feature of familicide [7,13], as high-risk periods for victimization may arise during instances of heightened familial tension, such as during divorce proceedings, custody battles, or following the issuance of protective orders [9], ending in murder.

As known, autopsy findings can provide significant forensic evidence, potentially confirming or refuting hypotheses about the circumstances of familicide [14]. Our study demonstrated a high level of severity of victim injuries, with the predominant number of injuries per victim being about 12, confirming that familicide is often characterized by overkill, signifying intense anger or rage [11,15,16]. From this count, asphyxiation and fire burns were excluded, as they are less frequent, but this may hint at the nature of the event, the level of planning involved, and the offender’s state of mind [10]. While in our cases, the use of a bladed weapon was the main manner of killing chosen by offenders, other studies have indicated that a large proportion of victims suffer blunt force trauma [7], and firearms appear to be the most common method used in familicide [8,13]. This may be explained by easily resulting in high lethality rates, but it was found less in our cases, due to Italian policies and restrictions for the possession of firearms.

The offenders in familicide cases are predominantly males, often middle-aged, who are either married or divorced [17]. Our cases showed the same trend. Many have a history of domestic violence and may be dealing with financial strain, unemployment, or other social stressors [7,15]. Employment status, in our cases, may have played a role in familicide. Only 19.35% of the victims and perpetrators were unemployed or engaged in occasional work. Retirees represented 16.13% of the victims, while self-employed victims accounted for another 16.13%. A smaller percentage of victims were employees (6.45%). Perpetrators often feel a profound sense of hopelessness, perceiving their act of familicide as a solution to their predicament [8]. In many cases, familicides are often premeditated acts driven by desperation, perceived familial dishonor, or perceived altruism [18,19]. Offenders may demonstrate certain behaviors before committing the act, such as isolation, emotional detachment, or notable changes in their routine [6,12]. A behavioral trajectory leading up to the act of familicide may be observed. Many perpetrators show escalating patterns of domestic violence or abusive behaviors, indicating that familicide often represents a severe escalation of ongoing domestic issues. This pattern underlines the necessity for early intervention in cases of domestic violence, which could help to prevent such an escalation [7,8,20]. Offenders may also be painted as individuals under considerable stress or strain, often related to financial hardships or relationship instability [6]. In our cases, economic motives were most frequent, representing 29.03%. These stressors are compounded by relationship conflicts, with a substantial proportion of perpetrators having a history of domestic violence [18,19,20]. The influences of societal constructs, particularly with respect to masculine identity and power dynamics, are also instrumental in understanding the driving forces behind these acts [19].

However, it is important to note that such behaviors may also be symptomatic of various mental health conditions, and thus not solely indicative of criminal intent [8]. Familicide offenders frequently exhibit signs of psychiatric disorders, particularly mood disorders and psychotic disorders [11]. Severe depressive disorders, bipolar disorders, and schizophrenia are commonly reported among individuals who commit familicide [14]. It is critical to recognize the role of untreated mental health disorders in such cases [13], as many perpetrators are dealing with chronic mental illness, often untreated or ineffectively managed [17,18]. Suicide frequently follows familicide, indicating a profound sense of desperation and hopelessness on the part of the perpetrator [19,20]. The act is sometimes viewed by the perpetrator as a twisted form of problem solving or an escape from an unbearable reality [8]. The prevalence of suicide in these cases underlines the importance of early mental health interventions and the availability of crisis support services. Additionally, autopsy findings from the offender can offer insights into their physical health and potential substance use, contributing to a comprehensive understanding of the case [6].

Several authors have concurred that familicide can be categorized based on the relationship between the victim and the offender, as they exhibit common characteristics in terms of behavioral profile and psychiatric findings. Liem et al. [12] presented an in-depth categorization of familicide types, reflecting the diversity and complexity of this phenomenon. Notably, they described two additional clusters beyond the more known “Despondent Fathers” and “Spousal Revenge”: the “Extended Parricide” and “Diffuse Conflict” clusters, underscoring the heterogeneity of familicide.

This heterogeneity within familicide echoes the multiplicity observed in other criminological phenomena, such as mass shootings and lone actor terrorism [13,17,18,19]. Despite the difference in the nature of these violent acts, they share the complexity of the motives driving them, emphasizing the importance of nuanced approaches to understanding each specific act of violence.

In terms of psychological aspects, Wilson et al. [6]’s exploration of lone actor terrorists’ mental health issues, although primarily contextualized within terrorism, could provide insights into some familicide cases. It would be interesting to further investigate whether similar psychopathologies, such as depression and suicidality, could be observed within the Despondent Fathers and Extended Parricide clusters.

The roles of revenge and power dynamics, highlighted in the work of Thomsen et al. [11] in the context of school shooters, could also resonate within the Spousal Revenge cluster of familicide. This underlines the overlapping motivations across different violent acts and emphasizes the need for individual-based approaches to understanding the motives underlying each cluster of familicide.

In the Italian context, Tosini D. [21] identified specific types of familicides based on the murderer’s motivations. One prominent type is the “doubly protective familicide”, where the act is perceived as a way of shielding the family from an impending catastrophic event. This could be driven by financial distress, health issues, or other personal anxieties. The act is seen as a twisted form of protection, where death is viewed as a refuge from perceived threats.

Another type highlighted is the “doubly punitive familicide.” Here, the perpetrator seeks to punish the partner for perceived wrongs, such as infidelity or estrangement. The children, in these cases, are often seen as contributors to the killer’s stress or believed to be siding with the mother, making them targets of the act.

The “indirectly punitive familicide” is another category where the children are victimized as an extension of the partner. In this scenario, the act of killing the children serves as a means of inflicting the ultimate pain and punishment upon the partner [22].

Another important kind of familicide that has aroused the interest of the scientific community is matricide. It has often been associated with a suspicion that the perpetrator might have a pathological mental state. The predominant psychiatric pattern among offenders is schizophrenia and other psychotic disorders, but substance abuse, mainly of alcohol, cocaine, and ketamine, has been reported in some cases [23]. Interviews with other family members may help in understanding and recognizing warning sings, such as isolation after a traumatic familiar event (such as separation of the parents), bizarre behavior, mood swings, threats, and violent behavior of a recent onset [24]. The significance of psychiatric evaluations in determining the criminal responsibility of matricide offenders is clear. Around 13 and 18% of offenders are deemed to be “Not Guilty by Reason of Insanity”, while about 25% have diminished criminal responsibility [23]. Regardless of the pathological substrate, matricide is often an extremely violent event: multiple sharp force injuries are the leading cause of death (55%), followed by blunt trauma (15%) and asphyxia (15%). Overkilling was reported in 12% of cases [23]. Lastly, according to Treglia et al. [25], the pandemic has caused significant socio-economic consequences, leading to an increased risk of violence within homes. The crisis caused by the COVID-19 pandemic, though comparable to the fallout of a natural disaster, has introduced distinct challenges. Extended social isolation, being confined to one’s home, and a deepening sense of disconnection from the community might collectively amplify the risk of developing psychotic symptoms and the escalation of domestic violence, especially in lower-income and conflictual social contexts.

## 5. Conclusions

While the diverse contexts and variables discussed in the files enriched our understanding of familicide, they also underlined the challenges in developing comprehensive predictive models and effective prevention strategies. Familicide, like other violent acts, is not a homogeneous phenomenon, and its heterogeneity must be central in any attempt to understand, predict, or prevent it.

## Figures and Tables

**Table 1:** Features of the victims and the offenders.

		Victim (31)	Offender (29)
Sex	Male, n (%)	14 (45.16%)	23 (79.31%)
	Female, n (%)	17 (54.84%)	6 (20.69%)
Age	Years, (Mean ± S.D.)	45.03 ± 23.75	45.63 ± 16.75
Victim/Perpetrator Relationship *	Parent, n (%)	5 (16.13%)	6 (19.35%)
	Offspring, n (%)	6 (19.35%)	5 (16.13%)
	Siblings, n (%)	4 (12.90%)	4 (12.90%)
	Spouses, n (%)	7 (22.58%)	7 (22.58%)
	Co-habitants, n (%)	3 (9.68%)	3 (9.68%)
	Others, n (%):	6 (19.35%)	6 (19.35%)
	Uncle, n (%)	1 (3.23%)	-
	Nephew, n (%)	-	1 (3.23%)
	Grandfather, n (%)	1 (3.23%)	-
	Grandchild, n (%)	-	1 (3.23%)
	Son-in-law, n (%)	3 (9.68%)	-
	Father-in-law, n (%)	-	3 (9.68%)
	Brothers-in-law, n (%)	1 (3.23%)	1 (3.23%)
Employment	Employee, n (%)	2 (6.45%)	3 (10.34%)
	Unemployed/casual worker, n (%)	6 (19.35%)	5 (17.24%)
	Pensioner, n (%)	5 (16.13%)	3 (10.34%)
	Self-employed/trader, n (%)	5 (16.13%)	2 (6.90%)
	Not available, n (%)	13 (41.95%)	16 (55.17%)

* In two cases of double homicide, the relationship of the offender with each of the victims was considered.

**Table 2:** Circumstantial and autopsy findings.

	Variable	
Number of injuries	Total per case (Mean ± S.D) * data	11.87 ± 19.94
Location of victim’s discovery	Private residence, n (%)	22 (70.97%)
	Street, n (%)	5 (16.13%)
	Hidden location, n (%)	2 (6.45%)
	Workplace, n (%)	2 (6.45%)
	Not available, n (%)	0 (%)
Type of injury mechanism	Firearm, n (%)	6 (19.35%)
	Bladed weapon, n (%)	15 (48.39%)
	Blunt object, n (%)	3 (9.68%)
	Asphyxia, n (%)	1 (3.23%)
	Fire, n (%)	1 (3.23%)
	Multiple methods, n (%)	5 (16.13%)
Location of injuries	Head, n (%)	18 (58.06%)
	Neck, n (%)	14 (45.16%)
	Chest, n (%)	20 (64.52%)
	Abdomen, n (%)	11 (35.48%)
	Upper limb, n (%)	14 (45.16%)
	Lower limb, n (%)	8 (25.81%)
Presence/absence of signs of struggle	Yes, n (%)	17 (54.84%)
	No, n (%)	14 (45.16%)

* Cases of asphyxia and fire usage were not considered.

**Table 3:** Psychiatric background of the offender and motive of the crime.

	Variable	
Presence of psychiatric pathology	Presence, n (%)	7 (24.14%)
Motive	Crime of passion, n (%)	4 (12.90%)
	Economic, n (%)	9 (29.03%)
	Psychiatric disorder, n (%)	6 (19.35%)
	Futile reasons, n (%)	5 (17.24%)
	Not Available, n (%)	7 (22.58%)

## Data Availability

Data are available on request due to restrictions, e.g., privacy or ethical The data presented in this study are available on request from the corresponding author.
